# Voltage-gated sodium channel expression in mouse DRG after SNI leads to re-evaluation of projections of injured fibers

**DOI:** 10.1186/1744-8069-10-19

**Published:** 2014-03-11

**Authors:** Cédric J Laedermann, Marie Pertin, Marc R Suter, Isabelle Decosterd

**Affiliations:** 1 Pain Center, Department of Anesthesiology, University Hospital Center and University of Lausanne, Lausanne 1011, Switzerland; 2 Department of Fundamental Neurosciences, University of Lausanne, Lausanne 1005, Switzerland

**Keywords:** Activating transcription factor 3 (ATF3), Dorsal root ganglia (DRG), Nerve injury, Neuropathic pain, Quantitative real time polymerase chain reaction (qRT-PCR), Sciatic nerve, Spared nerve injury (SNI), Spinal nerve ligation (SNL), Voltage-gated sodium channels (Na_v_s)

## Abstract

**Background:**

Dysregulation of voltage-gated sodium channels (Na_v_s) is believed to play a major role in nerve fiber hyperexcitability associated with neuropathic pain. A complete transcriptional characterization of the different isoforms of Na_v_s under normal and pathological conditions had never been performed on mice, despite their widespread use in pain research. Na_v_s mRNA levels in mouse dorsal root ganglia (DRG) were studied in the spared nerve injury (SNI) and spinal nerve ligation (SNL) models of neuropathic pain. In the SNI model, injured and non-injured neurons were intermingled in lumbar DRG, which were pooled to increase the tissue available for experiments.

**Results:**

A strong downregulation was observed for every Na_v_s isoform expressed except for Na_v_1.2; even Na_v_1.3, known to be upregulated in rat neuropathic pain models, was lower in the SNI mouse model. This suggests differences between these two species. In the SNL model, where the cell bodies of injured and non-injured fibers are anatomically separated between different DRG, most Na_v_s were observed to be downregulated in the L5 DRG receiving axotomized fibers. Transcription was then investigated independently in the L3, L4 and L5 DRG in the SNI model, and an important downregulation of many Na_v_s isoforms was observed in the L3 DRG, suggesting the presence of numerous injured neurons there after SNI. Consequently, the proportion of axotomized neurons in the L3, L4 and L5 DRG after SNI was characterized by studying the expression of activating transcription factor 3 (ATF3). Using this marker of nerve injury confirmed that most injured fibers find their cell bodies in the L3 and L4 DRG after SNI in C57BL/6 J mice; this contrasts with their L4 and L5 DRG localization in rats. The spared sural nerve, through which pain hypersensitivity is measured in behavioral studies, mostly projects into the L4 and L5 DRG.

**Conclusions:**

The complex regulation of Na_v_s, together with the anatomical rostral shift of the DRG harboring injured fibers in C57BL/6 J mice, emphasize that caution is necessary and preliminary anatomical experiments should be carried out for gene and protein expression studies after SNI in mouse strains.

## Background

Increased electrical activity is a major mechanism in the development of neuropathic pain following peripheral nerve injury. Spontaneous electrical discharges can originate from both injured and non-injured nerve fibers or from dorsal root ganglia (DRG) [1-7]. Voltage-gated sodium channels (Na_v_s) are key transmitters in cellular excitability [8], and are essential for pain transmission [9]. Na_v_s are heteromeric proteins composed of a large, pore-forming α-subunits and small β-auxiliary subunits [10,11]. Of the nine distinct channel isoforms described (Na_v_1.1 to Na_v_1.9), Na_v_1.5, Na_v_1.8 and Na_v_1.9 are resistant to tetrodotoxin (TTX). All isoforms, except Na_v_1.4 and Na_v_1.5, are expressed in DRG. Na_v_1.7 is the most highly expressed, TTX-sensitive isoform in rats [12-17]. It has been proposed that nerve-injury-mediated hyperexcitability results from the altered expression of Na_v_s [18-20]. In rats, these changes in Na_v_s expression occur in both injured and non-injured neurons [19,21-23]. In different experimental models of neuropathic pain in rats, the mRNAs of most Na_v_s were downregulated in the DRG [16,24-27] except for an increase of Na_v_1.3 transcript [16,27,28]. Na_v_s changes in mouse models of neuropathic pain have, however, not been investigated.

The various animal models of neuropathic pain, involving transection and/or ligation of nerves from the hind paw, exhibit different relations between injured and non-injured fibers. The first behavioral model of nerve injury was the complete sciatic nerve transection [29]. This model does not adequately reflect the partial nerve injuries observed in most patients with neuropathic pain, which also involves signals coming from intact sensory neurons [30]. Since then, models of partial injuries have been described which also allowed evoked behavioral testing of the hind paw. The L5 spinal nerve ligation (SNL) is an experimental neuropathic pain model which displays a clear separation between injured and non-injured cell bodies [31]. This model does not allow cross-talk between injured and non-injured cell bodies in L5 and L4 DRG, respectively. The spared nerve injury model (SNI) [32] involves the lesion of two terminal branches of the sciatic nerve, the common peroneal and tibial nerves, sparing the sural nerve, and inducing mechanical and thermal hypersensitivity in the latter territory. In this model, injured and intact nerves intermingle in the same DRG, which may allow cross-excitation between cell bodies [33,34] in addition to ephaptic cross-talk along nerve fibers [35]. Originally a rat model, the SNI model was later transposed and validated in mice [36]. To our knowledge, a careful characterization of injured and non-injured nerve fibers projecting into DRG has not been carried out in mice after SNI, and the assumption of neuroanatomical similarities between the two species—rats and mice—may not be correct [37].

In this study, we investigated the changes in Na_v_s transcription in mouse DRG following SNI and SNL surgery. To correlate Na_v_s expression to injury we also studied the projection of injured and intact fibers into the L3, L4 and L5 DRG after SNI.

## Results and discussion

### Expression of Na_v_s in mouse L4 and L5 DRG

**Figure 1:**
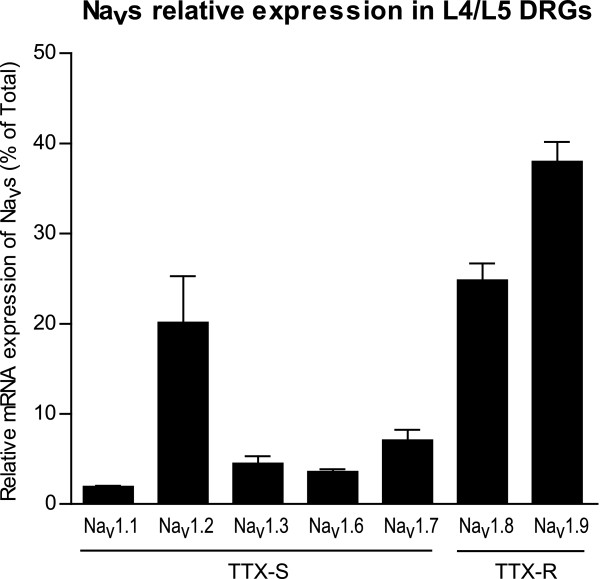
**Constitutive mRNA expression of Na**_**v**_**s isoforms in sham mouse DRG.** Constitutive levels of mRNA were determined using qPCR and normalized using GAPDH as a reference gene. Data are expressed as mean ± SEM, *n* = 4 samples (2 animals per sample).

**Figure 2:**
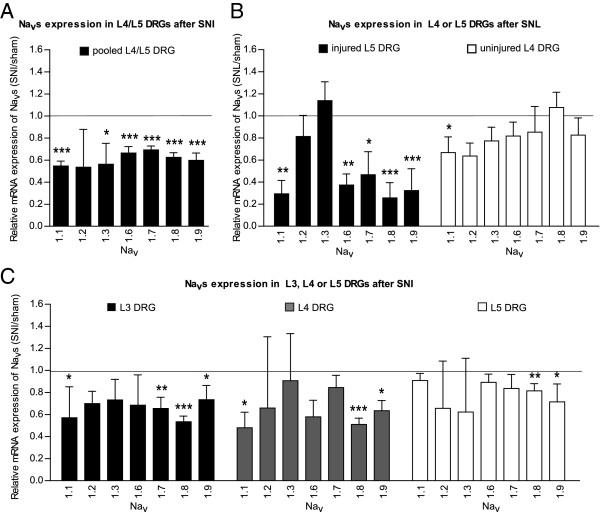
**SNI and SNL modulate Na**_**v**_**s mRNA expression in mouse DRG.** Transcription profile: **(A)** one week after SNI in pooled L4 and L5 DRG. Na_v_1.1, Na_v_1.3, Na_v_1.6, Na_v_1.7, Na_v_1.8 and Na_v_1.9 were downregulated after SNI. Only Na_v_1.2 remained unchanged. *p < 0.05, ***p < 0.001, Student’s *t* test. The bar graph represents the SNI/sham ratios.% changes in transcripts and *p-values* are found in Table 2. **(B)** One week after SNL in L4 and L5 DRG. In injured L5 DRG (black bars), Na_v_1.1, Na_v_1.6, Na_v_1.7, Na_v_1.8 and Na_v_1.9 were significantly lower. Na_v_1.2 and Na_v_1.3 were unchanged. In non-injured L4 DRG (white bars), only Na_v_1.1 was lower but Na_v_1.2, Na_v_1.3, Na_v_1.6, Na_v_1.7, Na_v_1.8 and Na_v_1.9 remained unchanged. *p < 0.05, **p < 0.01, ***p < 0.001, two-way ANOVA and *post hoc* Bonferroni tests. The bar graph represents the SNL/sham ratios for L4 and L5 DRG.% change and *p-values* are found in Table 3. **(C)** One week after SNI in separated L3, L4 and L5 DRG. Na_v_1.1 was significantly downregulated in L3 and L4. Na_v_1.2, Na_v_1.3 and Na_v_1.6 remained statistically unchanged in every DRG tested. Na_v_1.7 was only significantly downregulated in L3. Na_v_1.8 and Na_v_1.9 were downregulated in all three DRG. *p < 0.05, **p < 0.01, ***p < 0.001, two-way ANOVA and *post hoc* Bonferroni tests. The bar graph represents the SNI/sham ratios for L3, L4 and L5 DRG.% change and *p-values* are found in Table 4.

First, the level of expression of Na_v_s in the DRG of sham-operated mice was assessed using qRT-PCR (Figure 1). Constitutively, Na_v_1.2 is the most expressed TTX-sensitive isoform in mouse L4 and L5 DRG; this differs from rats, where this isoform is only faintly expressed [12,16,17]. It is noteworthy that significant variability in the expression for this isoform was observed (see Figure 2). Na_v_1.7, which is the main TTX-sensitive isoform expressed in rat DRG, was also well represented in mice, as it was the second most expressed TTX-sensitive isoform. Consistent with observations in rats, the two TTX-resistant isoforms—Na_v_1.8 and Na_v_1.9—were also highly expressed in mouse L4 and L5 DRG. The qRT-PCR products of all the Na_v_s isoforms were subcloned and sequenced, and, surprisingly, despite the careful design of specific *in silico* primers, some of the amplicons were indeed seen to be the cross-amplification products of other isoforms. In order to avoid artefactual results, all final primers used in this study (Table 1) were validated by sequencing the amplicons (see Methods). These results suggest that amplicons should always be sequenced when studying a highly conserved family of proteins such as Na_v_s.

**Table 1:** List of primers sequences

**Gene name**	**Primer sequence 5′-3′**	**Primer concentration**
GAPDH	(Fw) TCCATGACAACTTTGGCATTG	200 nM
	(Rev) CAGTCTTCTGGGTGGCAGTGA	
ATF3	(Fw) AGCTGAGATTCGCCATCCAGAA	200 nM
	(Rev) CTCGCCGCCTCCTTTTCCT	
Na_v_1.1	(Fw) AACAAGCTTGATTCACATACAATAAG	200 nM
	(Rev) AGGAGGGCGGACAAGCTG	
Na_v_1.2	(Fw) GGGAACGCCCATCAAAGAAG	100 nM
	(Rev) ACGCTATCGTAGGAAGGTGG	
Na_v_1.3	(Fw) AGGCATGAGGGTGGTTGTGAACG	300 nM
	(Rev) CAGAAGATGAGGCACACCAGTAGC	
Na_v_1.6	(Fw) AGTAACCCTCCAGAATGGTCCAA	200 nM
	(Rev) GTCTAACCAGTTCCACGGGTCT	
Na_v_1.7	(Fw) TCCTTTATTCATAATCCCAGCCTCAC	200 nM
	(Rev) GATCGGTTCCGTCTCTCTTTGC	
Na_v_1.8	(Fw) ACCGACAATCAGAGCGAGGAG	200 nM
	(Rev) ACAGACTAGAAATGGACAGAATCACC	
Na_v_1.9	(Fw) TGAGGCAACACTACTTCACCAATG	300 nM
	(Rev) AGCCAGAAACCAAGGTACTAATGATG	

### Downregulation of Na_v_s expression after SNI injury

Next, Na_v_s mRNA regulation after SNI in mice was analyzed. In order to reduce the number of animals necessary for experiments, and because DRG only contain scarce amounts of tissue, DRG are commonly pooled together. In procedures corresponding to those previously carried out in rats, mouse L4 and L5 DRG were pooled, as it was assumed that these would contain a mixture of the cell bodies from injured and non-injured fibers. In comparison to the sham-operated mice, SNI induced a significant downregulation of every isoform tested (Figure 2A and Table 2) except for Na_v_1.2. This downregulation may prove to be sustained as Na_v_1.7 (-33%, *p* = 0.019) and Na_v_1.8 (-38%, *p* = 0.007) were still significantly downregulated 6 weeks after SNI (data now shown). At the same time point, Na_v_1.3 downregulation had not reached significance (-24%, *p* = 0.43). The other isoforms were not tested.

**Table 2:** **Changes in transcriptional level of Na**_**v**_**s in pooled L4/L5 DRG after SNI**

	**% of modification (SNI/sham)**	***p-values *** **of treatment (SNI)**
DRGs	L4/L5	
Na_v_1.1	-45%	*p* < 0.001
Na_v_1.2	-46%	p = 0.155
Na_v_1.3	-44%	*p* = 0.011
Na_v_1.6	-34%	*p* < 0.001
Na_v_1.7	-31%	*p* < 0.0001
Na_v_1.8	-38%	*p* < 0.0001
Na_v_1.9	-40%	*p* < 0.0001

% of change of Na_v_s transcript in SNI as compared to sham in pooled L4/L5 DRG. Student’s *t* test to compare sham to SNI for every Na_v_s isoform.

These results highlighted an important difference between mice and rats: whereas Na_v_1.3 was upregulated in rats [16,28,38,39] after SNI, a downregulation of this isoform was observed in mice after SNI. Despite controversies about the role of sodium channels in neuropathic pain, the upregulation of Na_v_1.3 is commonly accepted as an important mechanism beyond neuropathic pain-associated hyperexcitability in rats [27,39]. This was recently confirmed by the gene’s knockdown in a rat model of nerve injury, which led to an attenuation of the nerve injury-induced neuropathic pain symptoms [40]. However, this study’s results indicated that Na_v_1.3 might be involved differently in mice, and this was corroborated by the normal development of neuropathic pain symptoms in Na_v_1.3 null mutant mice [41].

How a decrease in Na_v_s mRNA in the DRG could contribute to hyperexcitability remains subject to debate. A redistribution of the mRNA from the cell bodies to the sciatic nerve has been shown for Na_v_1.8, where this isoform can be translated and regain function [42]. In a previous paper, this study’s authors reported the interesting fact that following SNI in mice, Na_v_1.8 protein expression in the sciatic nerve increased [43]. Moreover, the level of mRNA does not necessarily correlate to amounts of protein, and further investigation will be necessary to unravel the physiological meaning of a decrease in Na_v_s transcripts in the DRG.

### Downregulation of Na_v_s expression after SNL injury

Because the L4 and L5 DRG contained adjacent injured and non-injured neurons after SNI, and in order to solely investigate the role of axotomy on Na_v_s expression, the following procedure to be performed was SNL. The L4 (non-injured) and L5 (injured) DRG were compared to their respective DRG in sham-operated mice.

A highly significant decrease in the mRNA expression of most of the Na_v_s isoforms was observed in the injured L5 DRG. Only the Na_v_1.2 and Na_v_1.3 isoforms remained unchanged in the injured L5 DRG (Figure 2B and Table 3). In contrast, most of Na_v_s isoform expressions in the non-injured L4 DRG remained unchanged in comparison to sham-operated mice, with the exception of a decrease of Na_v_1.1 mRNA.

**Table 3:** **Changes in transcriptional level of Na**_**v**_**s in injured (L5) and non-injured (L4) DRG after SNL**

	**% of modification (SNL/sham)**		***p-values *** **of treatment (SNL) for each DRG**		**Overall *p-value *** **of treatment (SNL)**
DRGs	L5	L4	L5	L4	
Na_v_1.1	-61%	-33%	**	*	*p* < 0.001
Na_v_1.2	-19%	-36%	ns	ns	*p* = 0.013
Na_v_1.3	+14%	-23%	ns	ns	*p* = 0.923
Na_v_1.6	-63%	-18%	**	ns	*p* = 0.004
Na_v_1.7	-53%	-15%	*	ns	*p* = 0.015
Na_v_1.8	-74%	+8%	***	ns	*p* = 0.003
Na_v_1.9	-78%	-17%	***	ns	*p* = 0.002

% of change of Na_v_s transcript in SNL as compared to sham in injured L5 and non-injured L4 DRG. 2–way ANOVA with independent measures to test for overall treatment effect and *post hoc* Bonferroni to test for statistical significance of treatment in each DRG (*p < 0.05, **p < 0.01 and ***p < 0.001).

The results for Na_v_1.6, Na_v_1.7, Na_v_1.8 and Na_v_1.9 seemed to indicate that their downregulation occurred exclusively in injured DRG. This was consistent with a previous study performed using a rat SNL model [44] where the authors reported a similar dichotomy for Na_v_1.8 and Na_v_1.9. However, this observation contrasts with the authors’ previous study carried in the rat after SNL [16], where small but significant increases of Na_v_1.6, Na_v_1.7, Na_v_1.8 and Na_v_1.9 were observed in the non-injured L4 DRG.

### Comparing Na_v_s expression modifications after SNI and SNL

Comparing observations of SNI and SNL results on the regulation of Na_v_1.6, Na_v_1.7, Na_v_1.8 and Na_v_1.9 further supported the fact that axotomy is responsible for their downregulation. A mixture of injured and non-injured DRG neurons revealed a ~40% decrease (L4/L5 SNI), and a DRG of enriched injured fibers revealed a decrease of ~65% (L5 SNL), in contrast to the lack of modification in DRG enriched in non-injured fibers (L4 SNL).

Na_v_1.1 was the only isoform to be downregulated in both non-injured L4 and injured L5 DRG, and this probably explains why it was the isoform which was most consistently lower in the SNI model (-65%). Even though cell bodies of injured and non-injured nerves are anatomically separated in different DRG, the decrease of Na_v_1.1 in the L4 DRG suggested possible cross-talk between injured and non-injured distal fibers in the SNL model.

Na_v_1.3 remains unchanged in the L4 and L5 DRG. This result seemed to contrast with the significant downregulation of Na_v_1.3 in the SNI experiment, and suggested that different types of lesion (either more distal or proximal) may have differing effects depending on the isoform. Furthermore, this lack of modification also contrasted with the authors’ previous study on the rat SNL model [16], further supporting differences between mice and rats.

### Regulation of Na_v_s in distinct DRG after SNI leads to reassessment of the innervation

**Figure 3:**
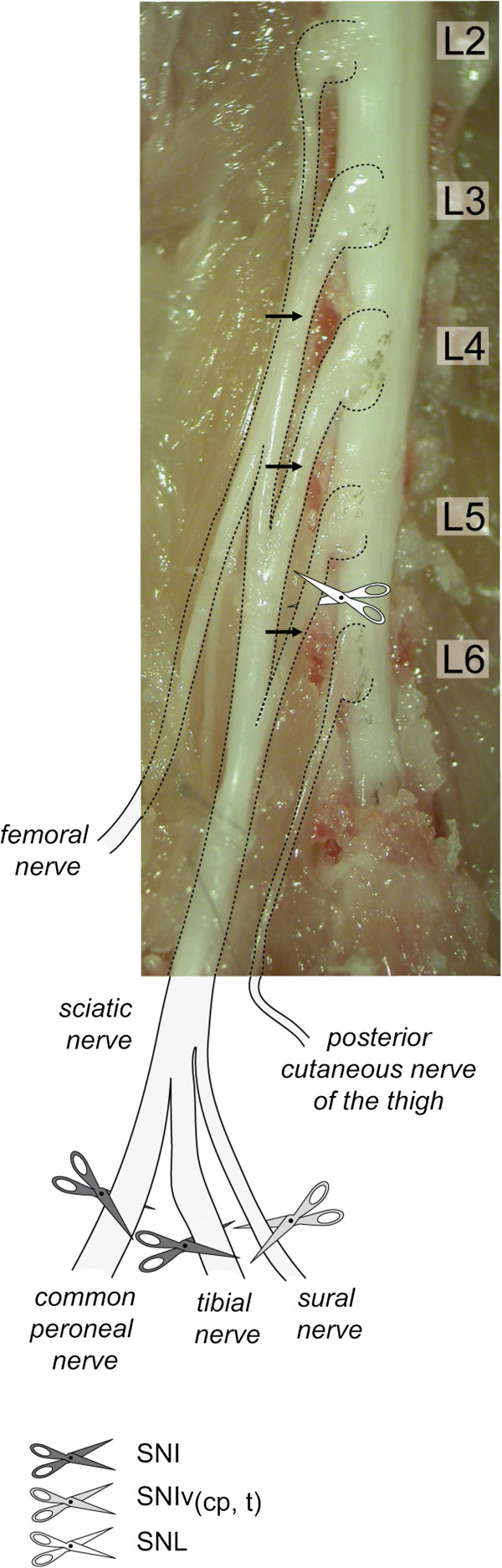
**Representative postero-lateral view of mouse DRG dissection.** In the photograph, the L3, L4 and L5 spinal nerves (black arrows), linked to L3, L4 and L5 DRG, respectively, are the main contributors of the sciatic nerve. L6 does not contribute to the sciatic nerve. Sites of SNI, SNIv_(cp,t)_ and SNL lesions are shown. SNI, Spared Nerve Injury; SNIv_(cp,t)_, Spared Nerve Injury variant, sparing common peroneal (cp) and tibial (t) nerves; SNL, Spinal Nerve Ligation.

To refine our analysis of SNI effects on Na_v_s regulation, we collected L4 and L5 DRG separately after surgery, instead of combining them. We also collected L3 DRG, because, as can be seen in the dissection procedure (Figure 3), and with regard to the differential anatomical relationships in mouse strains described by Rigaud *et al.*[37], these DRG were likely to provide fibers to the sciatic nerve. Na_v_1.1 mRNA was significantly lower in L3 and L4 DRG, and remained unchanged in L5 (Figure 2C and Table 4). Na_v_1.2, Na_v_1.3 and Na_v_1.6 mRNA expression remained unchanged across the three DRG as a whole, despite an observed trend to being lower in L3. Na_v_1.7 mRNA was significantly lower in L3 DRG, but remained statistically unchanged in L4 and L5. Na_v_1.8 mRNA was strongly downregulated in L3 and L4 DRG, and was also downregulated in L5 DRG, but to a minor extent. Na_v_1.9 mRNA was downregulated to a similar level in all three DRG.

**Table 4:** **Changes in transcriptional levels of Na**_**v**_**s in L3, L4 and L5 DRG after SNI**

	**% of modification (SNI/sham)**			**p-values of treatment (SNI) for each DRG**			**Overall p-value of treatment (SNI)**
DRG	L3	L4	L5	L3	L4	L5	
Na_v_1.1	-43%	-52%	-9%	*	*	ns	*p* = 0.002
Na_v_1.2	-30%	-34%	-34%	ns	ns	ns	*p* = 0.112
Na_v_1.3	-27%	-9%	-38%	ns	ns	ns	*p* = 0.113
Na_v_1.6	-32%	-42%	-11%	ns	ns	ns	*p* = 0.008
Na_v_1.7	-35%	-16%	-16%	**	ns	ns	*p* < 0.001
Na_v_1.8	-47%	-49%	-19%	***	***	**	*p* < 0.001
Na_v_1.9	-27%	-37%	-29%	*	*	*	*p* < 0.001

% of change of Na_v_s transcript in SNI as compared to sham in L3, L4 and L5 DRG independently. 2–way ANOVA with independent measures to test for overall treatment effect and *post hoc* Bonferroni to test for statistical significance of treatment in each DRG (*p < 0.05, **p < 0.01 and ***p < 0.001).

It was previously observed that Na_v_1.7, Na_v_1.8 and Na_v_1.9 were principally downregulated in injured fibers (Figure 2B) in the SNL model. The greater decrease of these three isoforms in the L3 DRG than in L5, further supports the possibility that L3 also harbors injured fibers following SNI surgery.

### Identification of L3, L4 and L5 DRG in C57BL/6 J mice

Segmentation of the lumbar vertebral column varies significantly between different strains of mice [45]. Rigaud *et al.* recently demonstrated that the vast majority of the DBA/2 J strain (97%) possessed five lumbar bony segments, whereas most of the C57BL/6 J strain (86%) possessed six [37]. Furthermore, these two strains also showed intra-species variability, and presented five or six segments, or even an asymmetrical fusion of the sixth lumbar vertebra. Because of this variability between strains, this study described the precise dissection procedure for harvesting the L3, L4 and L5 DRG in C57BL/6 J mice.

Figure 3 shows a representative photograph of the sciatic nerve, the L2 to L6 spinal nerves with their DRG, and spinal cord of a C57BL/6 J mouse after dissection. The sites of SNI, a SNI variant (sparing the common peroneal (cp) and tibial (t) nerves and noted as SNIv_(cp,t)_) [36], and SNL injuries are illustrated on the picture and on the drawn extensions of the sciatic nerve trifurcation into sural, common peroneal and tibial nerves. Following the sciatic nerve in the rostral direction leads to the first bifurcation heading to the L5 spinal nerve and to the branches leading to L4 and L3 DRG. Fibers from the sciatic nerve can be seen continuing towards the L3 DRG. Based on the dissection, it is likely that the L3 DRG also receive afferents from the femoral/saphenous nerve. Unlike in rats, none of the fibers in the sciatic nerve in mice originate from the L6 DRG; this seems to confirm that to find mouse DRG homologous to the rat, we must make a rostral shift [37].

### Injured fibers in the SNI model in mice project into L3 and L4

**Figure 4:**
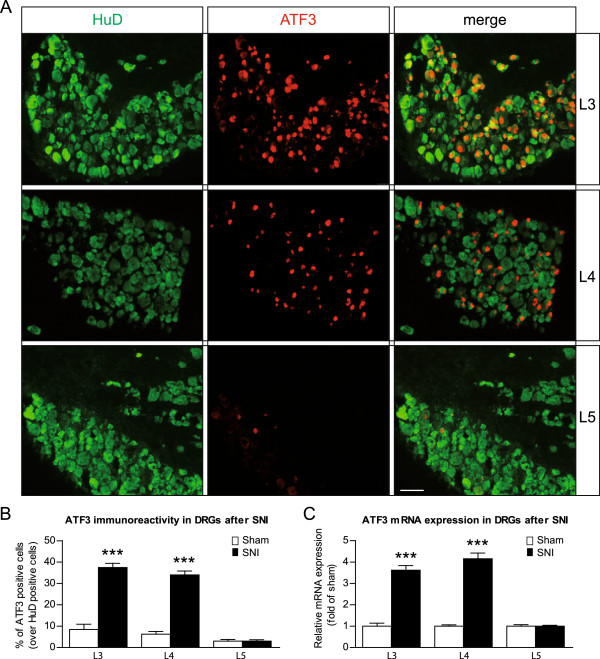
**ATF3 expression increases in mouse L3 and L4 DRG neurons after SNI, but not L5. (A)** Representative immunofluorescence showing that ATF3 (marker of injured neurons, red) was mostly up-regulated in L3 and L4 DRG neurons (HuD positive cells, green) after SNI. Scale bar = 50 μm. **(B)** Quantification of ATF3-immunoreactivity (IR) in L3, L4 and L5 DRG neurons one week after SNI or sham surgery. ATF3-IR was higher in L3 and L4 after SNI, but remained the same in L5 DRG. Data are expressed as mean ± SEM, *n* = 4 animals in each group. ***p < 0.001, two-way ANOVA and *post hoc* Bonferroni tests. **(C)** mRNA levels of ATF3 one week after SNI compared to sham surgery in L3, L4 and L5 DRG. ATF3 mRNA was higher in L3 and L4 after SNI, but not in L5. Levels of transcripts were first normalized to GAPDH as a reference gene, and then to sham for each DRG. Data are expressed as mean ± SEM, *n* = 3–4 animals in each group. ***p < 0.001, two-way ANOVA and *post hoc* Bonferroni tests. SNI, Spared Nerve Injury.

In rats, 98% of sciatic nerve fibers originate from the L4 and L5 DRG, whereas the somas of the saphenous nerve (part of the femoral nerve) fibers are located in the L3 DRG [46]. Therefore, the L4 and L5 DRG are those of interest in the SNI rat model. However, Rigaud *et al.* demonstrated that the functional and anatomical homologues of the rat L4 and L5 DRG were rather in the L3 and L4 DRG in mice [37]. This, together with the present study’s observation that L3 DRG showed a stronger downregulation of the Na_v_s transcript than L5, suggested that it may be necessary to reconsider which ganglia are likely to harbor the somas in the SNI mouse model. We consequently investigated the amount of injured fiber received by each ganglion after SNI. The expression of ATF3 was studied; it is a member of ATF/CREB family and a marker of axotomized neurons [47,48]. In sham-operated animals, ATF3-immunoreactivity (IR) was barely observable and reached a maximum of 8% for L3 (Additional file 1: Figure S1 and Figure 4B). Because naïve animals showed no IR for ATF3 (Additional file 1: Figure S1), it is likely that surgery itself activates ATF3 expression, as has already been proposed [49]. Seven days after SNI surgery, the percentage of ATF3-positive cells in L3 (37%) and L4 DRG (34%) was significantly higher than in sham-operation conditions (Figure 4A, B), yet in L5 the low percentage of ATF3-positive cells observed (3%) remained at sham levels. This result contrasts with the strong increase of ATF3 expression observed in L5 in rats after SNI [50], and clearly confirms that in mice, the cell bodies of most of the common peroneal and tibial injured fibers are located in L3 and L4 DRG rather than in L5.

The mRNA expression of ATF3 in the L3 to L5 DRG was also studied using qRT-PCR. This approach supported findings of a very significant increase of ATF3 in L3 and L4 DRG, but no change in expression in L5 (Figure 4C).

**Figure 5:**
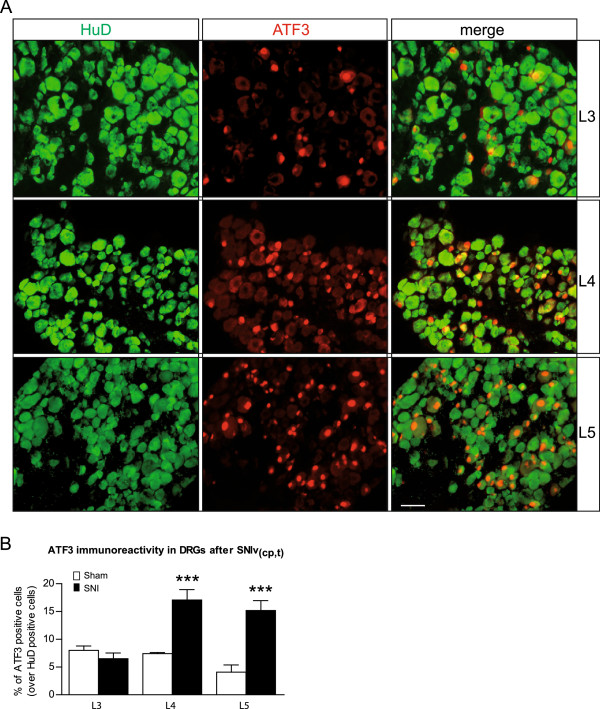
**ATF3 expression increases in mouse L4 and L5 DRG neurons after SNIv**_**(cp,t), **_**but not L3. (A)** Representative immunofluorescence showing that ATF3 (marker of injured neurons, red) was mostly upregulated in L4 and L5 DRG neurons (HuD positive cells, green) after SNIv_(cp,t)_. Scale bar = 50 μm. **(B)** Quantification of ATF3-immunoreactivity (IR) in L3, L4 and L5 DRG neurons one week after SNIv_(cp,t)_ or sham surgery. ATF3 was higher in L4 and L5 DRG when the sural nerve was injured, but not in L3. Data are expressed as mean ± SEM, *n* = 4 animals in each group. ***p < 0.001, two-way ANOVA and *post hoc* Bonferroni tests. SNIv_(cp,t)_, Spared Nerve Injury variant, sparing common peroneal (cp) and tibial (t) nerves.

So what is the relevance of the L5 DRG in the SNI mouse model? The above approach was used on the SNI variant, transecting only the sural nerve (SNIv_(cp,t)_) in order to investigate which ganglia the fibers from this nerve would project into. Sham surgery revealed 8%, 7% and 4% of ATF3-IR cells for L3, L4 and L5 DRG, respectively (Figure 5B), which was not different from sham-condition percentages of the traditional SNI. After SNIv_(cp,t)_, there was a significant increase of ATF3-IR cells in L4 (17%) and L5 (15%) DRG compared to sham conditions (Figure 5A and B). Conversely, the number of injured cells had not increased in L3 (7%), suggesting that the sural nerve originates in the L4 and L5 DRG.

**Figure 6:**
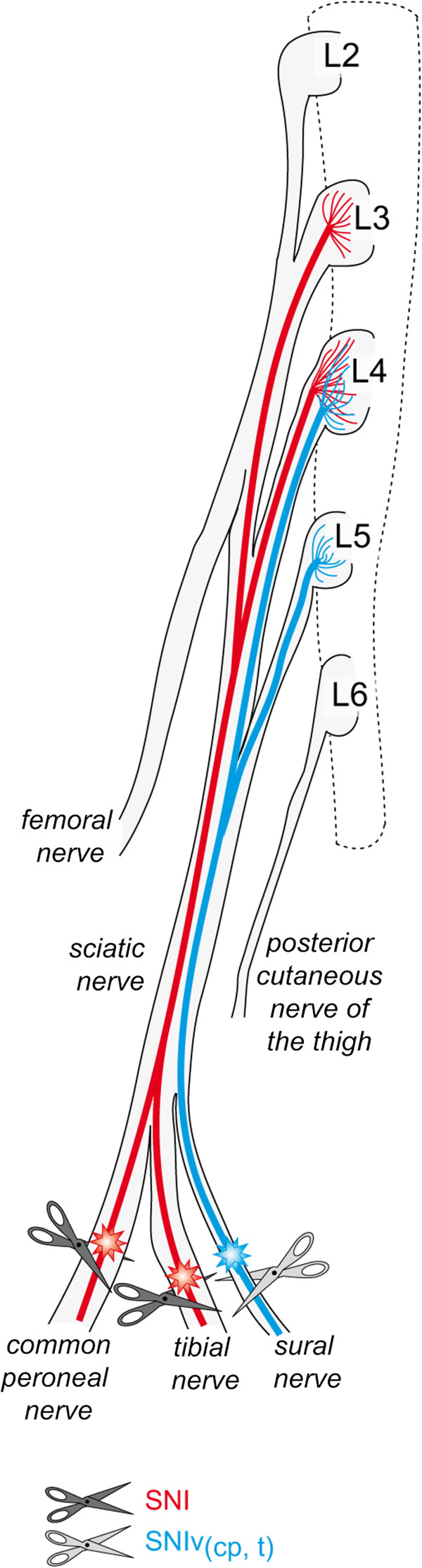
**Schematic view of sciatic nerve branches with projections of injured fibers into DRG.** The schematic view shows that common peroneal and tibial nerves predominantly originate in the L3 and L4 DRG (red fibers), while the sural nerve mainly originates from the L4 and L5 (blue fibers). SNI, Spared Nerve Injury; SNIv_(cp,t),_ Spared Nerve Injury variant, sparing common peroneal (cp) and tibial (t) nerves.

In summary, after SNI, the L3 DRG was comprised of a substantial proportion (~ 40%) of neurons from the injured common peroneal and tibial nerves, but none from the sural nerve. L4 DRG was constituted of neurons from the injured common peroneal and tibial nerves (~ 35%) and neurons from the sural nerve (~ 15%). Finally, the L5 DRG was comprised of no injured neurons from the injured common peroneal and tibial nerves after SNI, but did contain 15% of sural nerve neurons (Figure 6).

This demonstrated that when using the SNI mouse model, DRG should be pooled with caution because the L3, L4 and L5 DRG provided very different profiles of injured and non-injured neurons. It should be kept in mind that the mixture of neurons from all three individually taken DRG might affect or dilute the overall analysis and results. Furthermore, when performing behavioral pain tests in mice, and as Rigaud *et al.*[37] already proposed, L4 injury is more suitable for studying neuropathic pain-like hyperalgesia in the SNL model. Because this study aimed to investigate the modification of Na_v_s expression in DRG enriched in injured fibers, we did not re-perform SNL surgery on the L4 DRG.

## Conclusion

We showed that the expression of most Na_v_s mRNAs was lower in the L3 and L4 DRG after SNI in mice. Na_v_1.3 showed either a slight downregulation, or an absence of regulation, after SNI and SNL, which contrasted with the robust upregulation observed in rats. This inter-species difference should be further investigated in nerve-injury mouse models. Investigating Na_v_s expression in the L3, L4 and L5 DRG independently, lead to a re-evaluation of where injured neurons are projected after SNI. The injured common peroneal and tibial nerves projected into the L3 and L4 DRG, and the non-injured sural nerve projected into the L4 and L5 DRG in C57BL/6 J mice. This is of great importance when investigating nerve-injury mediated modifications in DRG after SNI in mice. We suggest that the L3 or L4 DRG should be harvested to target and enrichment in somas of injured fibers and L5 to enrich for the soma of non-injured fibers.

## Methods

### Surgery

All procedures were approved by the Canton of Vaud’s Committee on Animal Experimentation (Switzerland), in accordance with Swiss Federal Law on Animal Welfare and International Association for the Study of Pain guidelines [51].

The spared nerve injury (SNI) model of neuropathic pain was previously described in rats [32,52] and mice [36]. Briefly, adult C57BL/6 J mice (Charles River, L’Arbresle, France) were anesthetized with 1.5% isoflurane and after exposure of the sciatic nerve, the common peroneal and tibial nerves were ligated together with a 6.0 silk suture (Ethicon, Johnson and Johnson AG, Zug, Switzerland) and transected. In the SNI variant (SNIv_(cp,t)_) [36] the ligation and transection were performed on the sural nerve, leaving the common peroneal and tibial nerves intact. The incision was closed in distinct layers (muscle and skin). Sham surgery was performed similarly except for the nerve ligation and transection.

Spinal nerve ligation (SNL) surgery was adapted from the procedure described by Kim and Chung [31], and transposed to mice. Briefly, after skin and muscle incision the L5 transverse process of vertebra was exposed and carefully removed. The L4 and L5 spinal nerves were exposed and the L5 spinal nerve was tightly ligated. The incision was closed in distinct layers (muscle and skin).

### Dissection

Briefly, mice were terminally anesthetized with sodium pentobarbital (Esconarkon; Streuli Pharma AG, Uznach, Switzerland) and the *biceps femoris* muscle of the left thigh was incised. The *genus descendes* artery was used as a reference for the muscle incision, which lead to the exposure of the sciatic nerve and the trifurcation of the peripheral branches: common peroneal, tibial and sural nerves. The sciatic nerve was followed in the rostral direction, removing muscle tissue until reaching the vertebral column. Vertebral lamina, pedicles and spinous processes were trimmed away to expose the spinal cord and DRG. For the nomenclature of DRG, refer to Figure 3.

### Quantitative real-time reverse transcription PCR (qRT-PCR)

Ipsilateral DRG were rapidly dissected and collected in RNAlater solution (Qiagen, Basel, Switzerland). For SNI, 2 series of mice were used, one where the L4 and L5 were pooled together, as usually done (4 DRG pooled from 2 mice per sample), and one series where L3, L4 and L5 were dissected separately (8 DRG pooled from 8 mice per sample). For SNL, L4 and L5 DRG were consistently separated (2 DRG pooled from 2 mice per sample) as they represented non-injured and injured neurons, respectively. For all conditions tested, *n* = 3–4 / sample were used. mRNA was extracted and purified using a RNeasy Plus Mini Kit (Qiagen) and quantified using a RNA 6000 Nano Assay (Agilent Technologies AG, Basel, Switzerland). A total of 600 ng of RNA was reverse-transcribed for each sample using Omniscript Reverse Transcriptase Kit (Qiagen). Primer sequences and working concentrations for Na_v_s α-subunits, ATF3 and glyceraldehyde-3-phosphate dehydrogenase (GAPDH) can be found in Table 1.

We used GAPDH as a reference gene to normalize Na_v_s mRNA expression since it is stable between sham and SNI conditions (M-value = 0.30), taking into account the efficiencies of qPCR reaction. Gene-specific mRNA analyses were performed using the iQ SYBR-green Supermix (BioRad, Reinach, Switzerland) and the iQ5 real-time PCR detection system (BioRad). Only reactions with appropriate amplification and melting curves were analyzed. All samples were run in triplicate. Normalized transcripts were then expressed as a ratio of the level in SNI and SNL models to that in sham-operated mice. The bar graphs in Figures 2A, B and C represent these ratios for each isoform. Each qPCR product was sequenced to confirm the specificity of amplification. Briefly, qPCR products were first loaded on a low-melt agarose gel to confirm the size of the amplicon. Amplicons were then subcloned in a pGEM-T Vector System (Promega, Madison, WI, USA), and sent for sequencing using T7 promoter (Fasteris, Geneva, Switzerland). All qPCR products were validated as specific for each of the Na_v_s tested using the primers in Table 1.

### Immunofluorescence

One week after sham SNI or SNIv_(cp,t)_ surgery, animals were terminally anesthetized with sodium pentobarbital (Esconarkon), and then transcardially perfused with saline solution, directly followed by paraformaldehyde 4% diluted in phosphate buffered saline (PBS). The L3 to L5 DRG were dissected, post-fixed at 4°C for 90 min and then transferred in sucrose solution (20% sucrose in PBS) overnight. The following day, tissues were mounted in cryoembedding fluid (Tissue-Tek; Sakura Finetek, Zoeterwoude, Holland), frozen, cryosectioned in 12 μm-thick sections and thaw-mounted onto slides.

The rabbit anti-ATF3 antibody (1:300, Santa Cruz Biotechnology, Heidelberg, Germany) was used as the nuclear marker of injured neurons, and the goat anti-HuD antibody (Elav like proteins, 1:50, Santa Cruz Biotechnology) was used as the marker of total neuron numbers. Secondary antibodies were as follows: Cy3-conjugated anti-rabbit (1:400, Jackson ImmunoResearch, Suffolk, UK) for ATF3, and AlexaFluor 488-conjugated anti-goat (Molecular Probes, Basel, Switzerland) for HuD. Standard protocols for fluorescent immunohistochemistry were used. Sections of DRG were blocked for 30 min at room temperature (RT) with normal horse serum (NHS) 10% and PBS 1X-Triton X-100 0.3%. Primary antibodies were diluted in NHS 5% and PBS 1X-Triton X-100 0.1%, and incubated on sections overnight at 4°C. Slides were washed in PBS 1X and then incubated for 90 min at RT with the corresponding secondary antibody diluted in NHS 1% and PBS 1X-Triton X-100 0.1%. Slides were washed in PBS 1X and mounted in Mowiol medium (Calbiochem, Merck Millipore, Darmstadt, Germany).

Fluorescence was detected using an epifluorescence microscope (AxioVision, Carl Zeiss, Feldbach, Switzerland). Images were taken at 20× magnification, with the same parameters used for all experimental conditions. The complete DRG images were reconstructed by juxtaposing the different images using Photoshop CS4 software (11.0, Sun Microsystems, Redwood City, CA). Mean cell counts from each DRG were the average of 4 to 7 sections. Each first section was selected randomly, and the following ones were chosen every 72 m from the series of consecutive cut sections. Four animals were analyzed per condition. The percentage of injured neurons was expressed as the number of ATF3-IR neurons over the total number of cells (HuD-IR neurons). It should be noted that the percentage of ATF3-positive cells was probably a slight under-estimation of the actual proportion of injured cells because it represented the ratio of ATF3-positive cells over HuD-positive cells, which were counted independently to the presence or absence of the nucleus (one cell might have been counted twice in different stack).

### Statistical analysis

For the expression of Na_v_s after SNI, normalized transcripts were compared between sham surgeries versus SNI conditions using bilateral, unpaired Student’s *t* test. We used a two-way ANOVA with independent measures for the analysis of ATF3 expression and Na_v_s (each of them separately) in the L3, L4 and L5 DRG independently; one variable being the treatment (SNI or SNL), the other being the DRG. We used *post hoc* Bonferroni tests to assess whether the treatment (SNI or SNL) had a significant effect on the expression of each Na_v_ isoform. GraphPad Prism (version 5.01) was used to calculate statistics. This software does not provide exact *p-values* for *post hoc* Bonferroni tests and stars are shown for significance (*p < 0.05, **p < 0.01 and ***p < 0.001).

## Abbreviations

Navs: Voltage-gated sodium channels; DRG: Dorsal root ganglia; SNI: Spared nerve injury; SNL: Spinal nerve ligation; ATF3: Activating transcription factor 3; TTX: Tetrodotoxin; HuD: Elav-like proteins; IR: Immunoreactivity.

## Competing interests

The authors declare no competing interests.

## Authors’ contributions

CJL wrote the manuscript and supervised and designed the experimental approach. MP designed the experimental approach and performed SNI, SNIv_(cp,t)_, immunofluorescence, cell counting and qPCR. M.R.S analyzed data and corrected the manuscript. ID supervised experimental approach and corrected the manuscript. All authors read and approved the final manuscript.

## Supplementary Material

Additional file 1: Figure S1 Sham surgery induces ATF3 expression in mouse L3, L4 and L5 DRG neurons. Representative immunofluorescence showing that ATF3 immunofluorescence was not observable in naïve animals (only L4 is shown). Conversely, ATF3-IR was induced in L3, L4 and L5 DRG after sham surgery. Scale bar = 50 μm. Click here for file
