# Supplementary figures and images for: Voltage-gated sodium channel expression in mouse DRG after SNI leads to re-evaluation of projections of injured fibers
Source: Mol Pain. 2014 Mar 11;10:19. doi: 10.1186/1744-8069-10-19 (PMC4007621; doi:10.1186/1744-8069-10-19)

sham

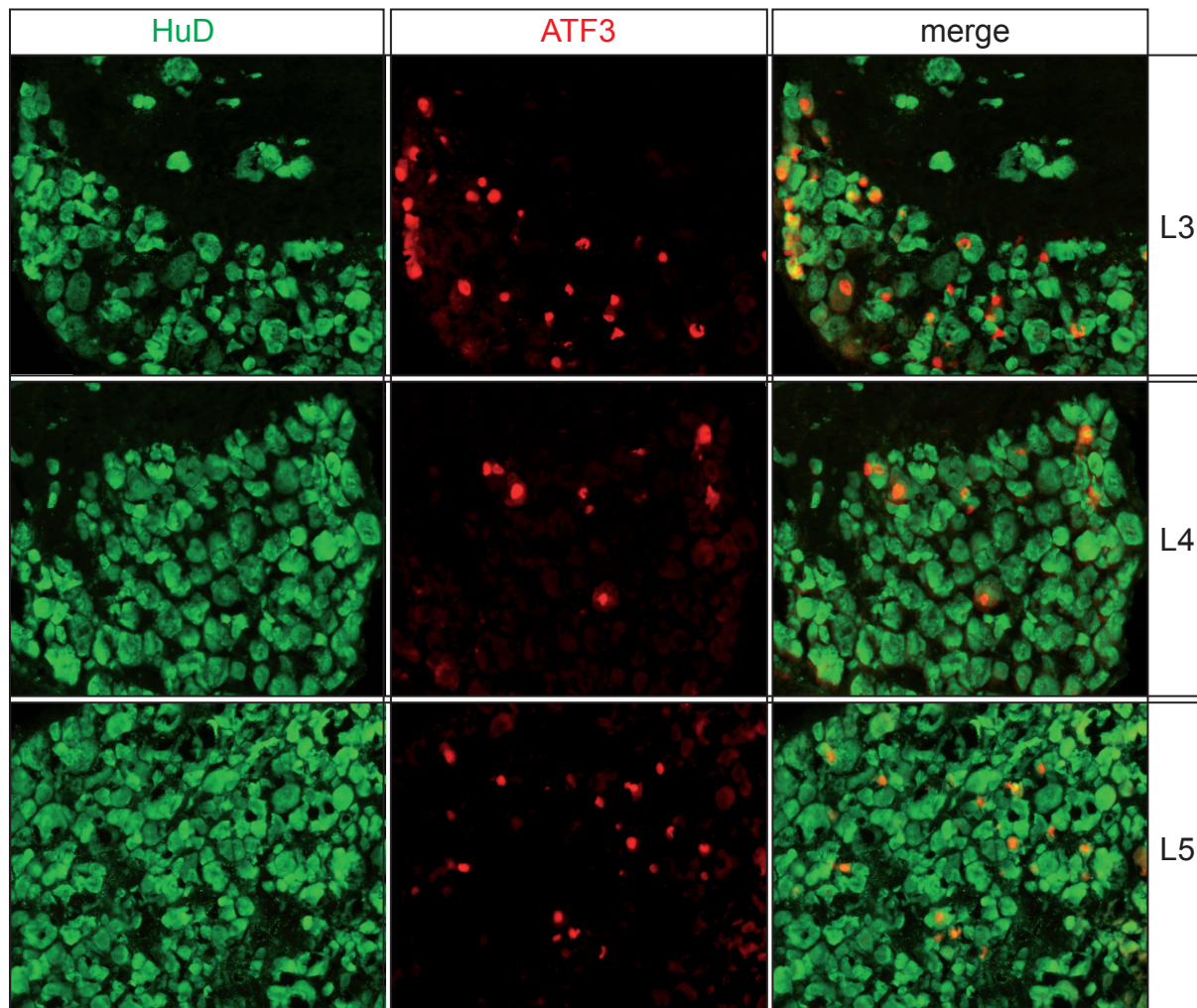

naïve

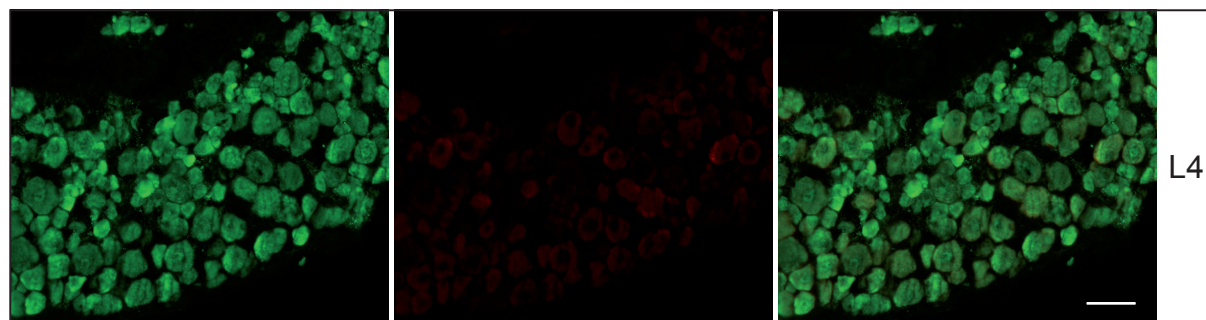

Supplemental Figure 1

Supplement: Additional file 1: Figure S1 — Sham surgery induces ATF3 expression in mouse L3, L4 and L5 DRG neurons. Representative immunofluorescence showing that ATF3 immunofluorescence was not observable in naïve animals (only L4 is shown). Conversely, ATF3-IR was induced in L3, L4 and L5 DRG after sham surgery. Scale bar = 50 μm. [file 1744-8069-10-19-S1.pdf]
